# Decreased Plasma IGF-1 Is Associated with Cortical Atrophy, but Not Concomitant Cerebrovascular Disease in Alzheimer’s Dementia

**DOI:** 10.3390/biom16091248

**Published:** 2026-08-28

**Authors:** Amelia T. Y. Yam, Yuek Ling Chai, Saima Hilal, Cai Yuan, Vincent C. T. Mok, Narayanaswamy Venketasubramanian, Boon Yeow Tan, Ming Ann Sim, Mitchell K. P. Lai, Christopher P. Chen, Joyce R. Chong

**Affiliations:** 1 Department of Pharmacology, Yong Loo Lin School of Medicine, National University of Singapore, Singapore 117600, Singapore; 2 Memory Aging and Cognition Centre, National University Health System, Singapore 117600, Singapore; 3 Healthy Longevity Translational Research Programme, Yong Loo Lin School of Medicine, National University of Singapore, Singapore 117456, Singapore; 4 Saw Swee Hock School of Public Heath, National University of Singapore, Singapore 117597, Singapore; 5 Division of Neurology, Department of Medicine and Therapeutics, The Chinese University of Hong Kong, Shatin, Hong Kong SAR, China; 6 Raffles Neuroscience Centre, Raffles Hospital, Singapore 188770, Singapore; 7 Department of Medicine, St Luke’s Hospital, Singapore 659674, Singapore; 8 Department of Anaesthesia, National University Health System, Singapore 119074, Singapore; 9 Wisconsin Alzheimer’s Disease Research Center, School of Medicine and Public Health, University of Wisconsin-Madison, Madison, WI 53792-2420, USA

**Keywords:** Alzheimer’s disease, insulin-like growth factor 1, biomarker, neurodegeneration, cerebrovascular disease

## Abstract

Dysregulated insulin signaling in the brain has been linked to cognitive impairment and dementia. Insulin-like growth factor 1 (IGF-1) is a peptide growth hormone crucial for neurogenesis and neuroprotection. Findings regarding potential involvement of IGF-1 in dementia have been conflicting, and the status of IGF-1 in clinical cohorts with Alzheimer’s disease (AD) and concomitant cerebrovascular disease (CeVD) burden is unknown. A Singapore-based memory clinic cohort consisting of 46 non-cognitively impaired (NCI), 101 with cognitive impairment, no dementia (CIND) and 81 AD dementia subjects underwent plasma IGF-1 measurements and neuroimaging assessments for association analyses of peripheral IGF-1 with regional brain volumes, as well as with neuroimaging CeVD markers (lacunes, cerebral microbleeds, white matter hyperintensities). Plasma IGF-1 levels were significantly lower in AD compared to NCI and CIND participants (both *p* < 0.001). Plasma IGF-1 was significantly associated with smaller hippocampal (*p* = 0.035), amygdala (*p* = 0.024), parietal lobe (*p* = 0.029), and frontal lobe (*p* = 0.002) volumes. In contrast, plasma IGF-1 did not associate with CeVD markers after covariate adjustments. Our findings suggest that plasma IGF-1 may be a blood-based biomarker for reduced brain volumes, while having no direct role in CeVD pathophysiology.

## 1. Introduction

Alzheimer’s disease (AD) is the most common type of neurodegenerative dementia, characterized by cortical β-amyloid (Aβ) plaque deposition and neurofibrillary tangles (NFTs). As the disease progresses, accumulative neuronal death and dysfunction lead to brain atrophy and cognitive impairment. Furthermore, while cerebrovascular disease is recognized as a distinct process which can lead to vascular dementia (VaD, as part of the spectrum of vascular cognitive impairment), there is increasing evidence that cerebrovascular disease may be a common co-pathology in patients with AD [1,2], which in turn contributes to AD progression, including the exacerbation of cognitive impairment and dementia severity [3,4]. Additionally, atherosclerosis of large vessels like the Circle of Willis has been reported to be associated with more severe cortical and hippocampal atrophy [5], while associations between cerebrovascular disease (CeVD) subtypes (such as white matter lesions, lacunar infarcts, cerebral microbleeds) and cortical atrophy are less clear. Importantly, whilst peripheral biomarkers of AD and VaD have been widely investigated [6], studies into biomarkers which can differentiate AD- vs. CeVD-associated processes in patients who manifest concomitant disease remain scarce.

There has been growing interest in the potential roles that insulin signaling dysregulation may play in the pathogenesis of cognitive impairment and dementia [7,8,9,10,11], where insulin-like growth factor 1 (IGF-1) is a key player in the insulin signaling axis. IGF-1 is a peptide hormone that is structurally similar to insulin but produced primarily by hepatocytes [12]. It is secreted in response to changes in growth hormone (GH) levels in the body and as such, IGF-1 concentrations peak at the age of puberty and decline progressively with age [13,14]. However, IGF-1 can also be found in neurons independently of GH [15,16], where it has key roles in neurogenesis and synaptogenesis [17,18]. In animal models, IGF-1 knockdown showed reduced adult hippocampal neurogenesis and impaired spatial memory [19,20]. Conversely, overexpression of IGF-1 in animal models increases brain volumes [21]. Recent clinical studies on the role of IGF-1 in neurodegeneration have shown complementary results, where a few studies reported associations between increased serum IGF-1 in AD patients and better cognition outcomes [22,23,24], while others have reported lower IGF-1 levels in AD patients [25,26], which were associated with lower brain volumes [27,28].

Reduced IGF-1 has also been linked to several vascular disease factors, including increased risks of ischemic stroke [29], hypertension [30], cardiovascular diseases [31,32,33], diabetes mellitus [34] and metabolic syndrome [35]. However, it is unclear if peripheral IGF-1 is associated with specific CeVD subtypes. In this study, we measured plasma IGF-1 in a Singaporean memory clinic cohort, and studied its associations with neuroimaging assessments of brain atrophy and CeVD.

## 2. Materials and Methods

### 2.1. Study Population

Details of the longitudinal study from which the participants of the present study are derived (n = 228, recruited between 2010 and 2013) have been previously described [36]. Briefly, informed consent was obtained from participants or their next-of-kin prior to recruitment into the study with approval from the Singapore National Healthcare Group Domain-Specific Review Board (2010/00017 and 2018/01098) for the collection of detailed medical and drug histories, clinical evaluations, neuroimaging, cognitive assessments (Mini-Mental State Examination (MMSE, score ranges 0–30, with higher scores indicating better function)) and Clinical Dementia Rating-Sum of Boxes (CDR-SB, score ranges 0–18, with higher scores indicating greater cognitive and functional impairment), as well as blood draw for plasma IGF-1 measurements.

### 2.2. Clinical Diagnoses of Cognitive Impairment and Dementia

Diagnoses of all participants were made at consensus meetings of study clinicians and neuropsychologists as previously described [36]. Briefly, participants with no cognitive impairment (NCI) had no objective cognitive impairment based on formal neuropsychological assessments, including a neuropsychology battery (Supplementary Table S1). Participants with subjective cognitive complaints were included in the NCI group as well. Participants with cognitive impairment no dementia (CIND) did not meet the Diagnostic and Statistical Manual of Mental Disorders, Fourth Edition (DSM-IV) diagnostic criteria for dementia but had impairment in at least one cognitive domain on a neuropsychological test battery as previously described [37]. Clinical diagnoses of AD dementia were made according to the DSM-IV criteria as well as the NINCDS-ADRDA criteria [38].

### 2.3. Assessment of Other Risk Factors

Risk factors (hypertension, hyperlipidemia, diabetes mellitus and cardiovascular diseases) were obtained through detailed questionnaires, clinical interviews and were classified in this study as present or absent, based on past medical history or usage of medication for the treatment of the condition. Hypertension was defined as systolic blood pressure of ≥140 mmHg and/or diastolic blood pressure of ≥90 mmHg or a history of use of anti-hypertensive medication. Hyperlipidemia was defined as having total cholesterol levels of ≥4.14 mM or a history of use of lipid-lowering medication. Diabetes mellitus was defined as glycated hemoglobin (HbA1c) of ≥6.5% or a history of use of diabetic medication. Cardiovascular disease was defined as having a positive history of atrial fibrillation, congestive heart failure and/or myocardial infarction. Positive *APOE4* (ε4 variant of the apolipoprotein E gene) status was also assessed as previously described [39] and defined as having at least one ε4 allele.

### 2.4. Neuroimaging

Magnetic resonance imaging (MRI) scans were performed on a 3T Magnetom Trio Tim scanner (Siemens Healthineers AG, Forchheim, Germany) using a 32-channel head coil at the Clinical Imaging Research Center, National University of Singapore. The sequences included T1-weighted, fluid attenuated inversion recovery (FLAIR), T2-weighted, and susceptibility-weighted imaging sequences (see Supplementary Data S1) as described previously [40].

#### 2.4.1. Brain Atrophy MRI Markers

**Figure 1:**
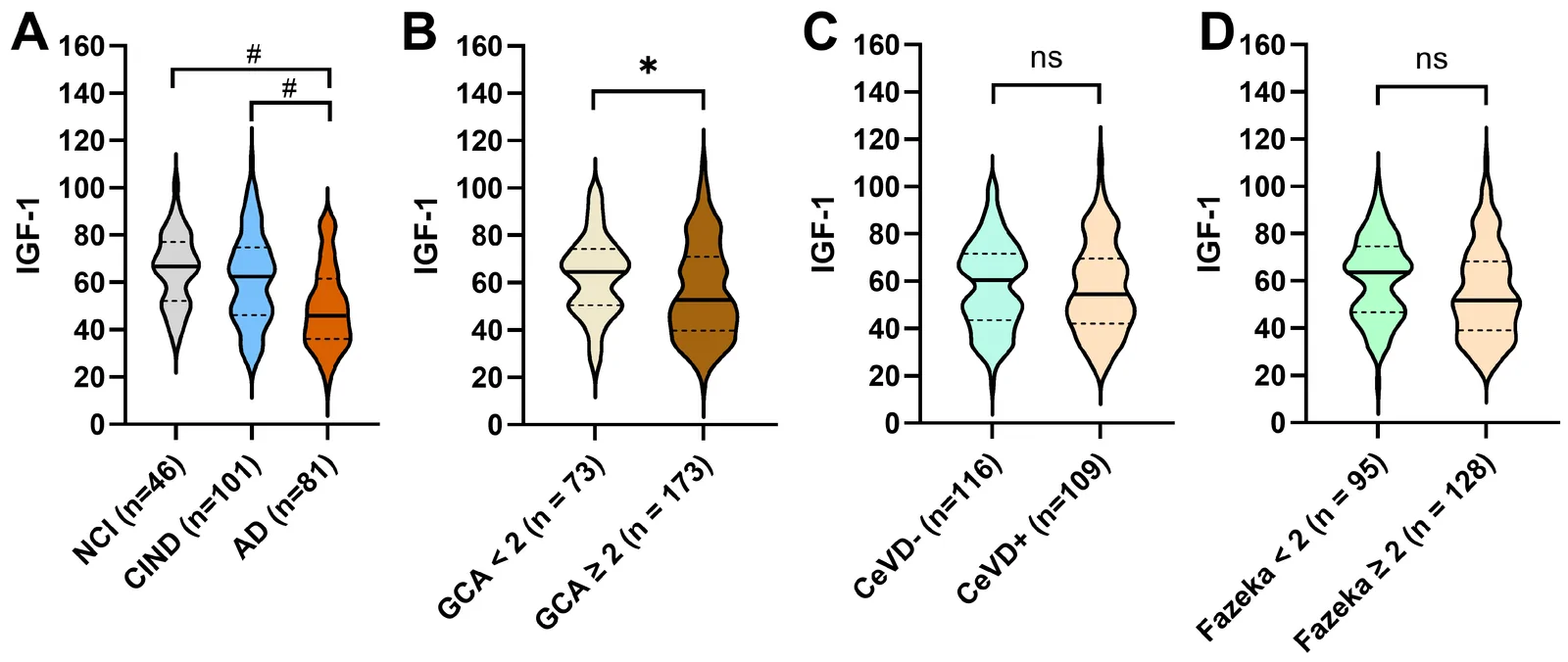
Plasma IGF-1 concentrations (in ng/mL) across diagnostic groups based on (**A**) clinical diagnosis; (**B**) presence of significant global cortical atrophy (defined as GCA scores ≥ 2); (**C**) presence of significant CeVD (see Section 2.4.2 above); and (**D**) presence of significant WMH (defined as Fazekas scores ≥ 2), depicted in violin graphs with the middle solid lines denoting median values, while top and bottom dotted lines represent respective quartiles. Abbreviations: AD, Alzheimer’s Disease; CeVD, cerebrovascular disease; CIND, cognitively impaired no dementia; NCI, no cognitive impairment. ^#^ *p* < 0.05 (Kruskal–Wallis ANOVA with *post hoc* Dunn–Bonferroni’s tests); * *p* < 0.05 (Mann–Whitney U tests).

Initial analysis consisted of global cortical atrophy based on dichotomization of GCA scores (<2, none to mild vs. ≥2, moderate to severe, see Figure 1) [41]. An automated segmentation procedure at the Department of Medical Informatics, Erasmus University Medical Center, the Netherlands (FreeSurfer, v.5.1.0), and subsequently a tissue classification algorithm [42] was used to quantify global cortical thickness, which was measured as the shortest distance between the gray/white matter boundary and pial surface of each vertex. All MRI scans were also processed with AccuBrain^®^ IV 2.0 (BrainNow Medical Technology Ltd., Hong Kong SAR, China), a fully automated software for brain volumetric segmentation and quantification [43,44] to obtain regional brain volumes (hippocampal, amygdala, temporal lobe, parietal lobe, frontal lobe and gray matter).

#### 2.4.2. Cerebrovascular Disease MRI Markers

Detailed descriptions of visual gradings of CeVD markers, namely white matter hyperintensities (WMH), lacunes and cerebral microbleeds (CMBs), together with cortical infarcts, can be found in Supplementary Data S1. Presence of significant WMH was also assessed based on dichotomization of Fazekas scores (<2, absent or only punctate foci vs. ≥2, beginning confluent or large confluent areas in deep white matter regions; see Figure 1) [41]. Furthermore, WMH was graded using the Age-Related White Matter Changes (ARWMC) scale [45], which was also dichotomized (≥8, a significant burden of white matter disease). Clinically significant cerebrovascular disease (CeVD+) was defined as the presence of cortical infarcts and/or ≥2 lacune, and/or confluent WMH (ARWMC ≥ 8) in at least 2 brain regions, as previously proposed [46].

### 2.5. Blood Biomarker Measurements

Non-fasting blood was collected from study participants and processed by centrifugation at 2000× *g* at 4 °C for 10 min for plasma extraction and stored at −80 °C until use. Plasma IGF-1 was measured by a quantitative sandwich immunoassay technique (Quantikine, Cat number: DG100B, R&D Systems Inc., Minneapolis, MN, USA) in accordance with the manufacturer’s instructions.

### 2.6. Statistical Analyses

Statistical analyses were performed using IBM SPSS Statistics version 26 (IBM Co., Armonk, NY, USA). Normally distributed continuous data are presented as mean ± standard deviation (SD), while skewed continuous data are presented as median (interquartile range, IQR). Group comparisons of continuous demographic variables were performed using independent *t*-tests and one-way analysis of variance (ANOVA) with *post hoc* Bonferroni tests for normally distributed data, and non-parametric Mann–Whitney U tests or Kruskal–Wallis test with Dunn’s procedure for skewed distributed data. For categorical variables, chi-square tests were used. Correlation analyses were performed using Spearman’s rank correlations. Negative binomial regression models were used for counts of cortical infarcts, lacunes and CMBs. Binary logistic regression models were used for ARWMC scores. All models were adjusted for age, sex, education, *APOE4* carrier status, hypertension, hyperlipidemia, diabetes and cardiovascular diseases. Volumetric measures were further adjusted for total intracranial volume. To account for multiple comparisons across regional MRI outcomes, *p*-values were adjusted using the Benjamini–Hochberg false discovery rate (FDR) procedure. Significance was set at *p* < 0.05.

## 3. Results

### 3.1. Participant Characteristics

Table 1 shows the baseline demographic characteristics of the 228 study participants. Among them, 46 (20.2%) were NCI, 101 (44.3%) were CIND and 81 (35.5%) were AD dementia participants. Compared to NCI, CIND and AD dementia participants were older and less educated. The prevalence of hypertension and diabetes was higher in AD dementia participants compared to NCI participants. No significant difference was observed in APOE ε4 carrier frequency, prevalence of hyperlipidemia and cardiovascular diseases among groups. Plasma IGF-1 levels were lower in female participants, and negatively correlated with age (Spearman’s *rho* = −0.429), but positively correlated with education (*rho* = 0.292). There was no significant difference in plasma IGF-1 levels in those with hypertension, hyperlipidemia, diabetes and cardiovascular diseases.

### 3.2. Plasma IGF-1 Changes Across Diagnostic Groups

When compared across the clinical diagnostic groups, a significant decrease in plasma IGF-1 was observed only in AD participants (mean [SD] = 49.2 [17.1] ng/mL) compared to the NCI (65.6 [15.1] ng/mL) and CIND (61.2 [19.1] ng/mL) groups (Figure 1A). There was no significant difference between NCI and CIND participants (*p* = 0.491). Plasma IGF-1 was also lower in participants with significant global cortical atrophy (GCA) (Figure 1B). In contrast, IGF-1 levels were not significantly altered when participants were segregated either by CeVD− vs. CeVD+ (Figure 1C) or specifically by presence vs. absence of significant WMH (Figure 1D).

### 3.3. Associations Between Plasma IGF-1 and MRI Markers of Brain Atrophy

Following the GCA analysis above, we performed correlation analyses with each brain region listed in Supplementary Table S2. All regional brain volumes analyzed were positively correlated with plasma IGF-1, with *rho* values ranging from 0.327 to 0.389 (all *p* < 0.001). Furthermore, we performed univariate linear regression of quantitative regional brain volumes adjusted for covariates. Table 2 shows linear regression models with significant associations with plasma IGF-1 after adjusting for age, sex, education and total intracranial volume (Model 1), and additionally for *APOE4* status, hypertension, hyperlipidemia, diabetes mellitus and cardiovascular diseases (Model 2): hippocampal volume (β = 0.956, *p* = 0.035), global cortical thickness (β = 0.133, *p* = 0.016), amygdala volume (β = 0.504, *p* = 0.024), parietal lobe volume (β = 8.336, *p* = 0.029), frontal lobe volume (β = 12.584, *p* = 0.048) and gray matter volume (β = 39.486, *p* = 0.016). Only temporal lobe volume (β = 8.638, *p* = 0.060) failed to reach statistical significance after Model 2 adjustments. Following Benjamini–Hochberg FDR correction for multiple comparisons, all six significant associations remained statistically significant (FDR-adjusted *p* < 0.05).

### 3.4. Associations Between Plasma IGF-1 and MRI Markers of CeVD

We initially showed that plasma IGF-1 levels did not significantly differ between CeVD− and CeVD+ participants (see Figure 1). We then proceeded to assess IGF-1 correlations with CeVD subtypes, and found that cerebral microbleed (CMB) count (*rho* = −0.095), lacune count (*rho* = −0.032) and cortical infarct count (*rho* = −0.046) were not correlated to plasma IGF-1 levels (all *p*-values > 0.05). ARWMC scores for WMH was negatively correlated with IGF-1 (*rho* = −0.168), but no associations were found for ARWMC or other CeVD types on regression analyses adjusted for covariates (see Table 3).

### 3.5. Sensitivity Analyses

As this study included participants with larger cortical infarcts as well as small vessel diseases, sensitivity analyses to assess the potential effects of larger lesions were performed by excluding participants with large structural lesions (n = 7) on demographic variables (Supplementary Table S3), diagnostic group differences (Supplementary Figure S1), regression analyses of brain atrophy measures (Supplementary Table S4) and CeVD markers (Supplementary Table S5), which showed that all results and conclusions remained unchanged, except for the loss of statistical significance for the association between plasma IGF-1 and frontal lobe volume.

### 3.6. Associations Between Plasma IGF-1 and Cognition

Given the strong association observed between IGF-1 and reduced brain volumes, as well as the established association between brain atrophy and cognitive impairment, we examined whether IGF-1 was associated with cognitive performance. Supplementary Table S6 shows that IGF-1 was positively correlated with baseline MMSE scores (*rho* = 0.245) and negatively correlated with CDR-Sum of Boxes (CDR-SoB) scores (*rho* = −0.277).

## 4. Discussion

This study on a Singapore-based cohort with comprehensive neuroimaging showed decreased plasma IGF-1 levels in AD dementia patients compared to NCI controls and CIND, corroborating a number of previous studies [25,26,47]. The dysregulation in the IGF-1 signaling axis has been shown to mediate the pathogenesis of AD [48], where IGF-1 deficits disrupt glucose metabolism in the brain, subsequently culminating in neuroinflammation [49], neuronal death and neurodegeneration [50]. Decreased IGF-1 levels have also been linked to worse prognosis in other neurodegenerative diseases such as Parkinson’s disease [51] and multiple sclerosis [52]. In parallel, adults with growth hormone disorders have demonstrated reduced IGF-1 and altered cortical thickness [53].

Our findings of significant associations between lower plasma IGF-1 levels and smaller hippocampal volume as well as cortical thinning are corroborated by prior studies [27,28,47,54]. Whilst hippocampal atrophy is a prominent feature in AD [55,56,57,58,59], accumulating evidence suggests that acceleration of brain volume loss occurs at different times relative to symptom onset [60]. This study is, to the best of our knowledge, the first to expand findings of IGF-1 associations with multiple other brain regions, and the positive association with gray matter volume and lobar volumes support the hypothesis that lower IGF-1 levels are related to more widespread structural brain changes beyond the hippocampus. Interestingly, plasma IGF-1 decreases were significantly associated with amygdala atrophy, which previous studies have observed in early stages of AD [59,61,62,63]. The amygdala atrophy pattern is predictive of conversion to dementia [64,65], although not as strongly as hippocampal atrophy [66]. Our findings further demonstrated an association between lower plasma IGF-1 levels and reduced parietal lobe volumes, a region increasingly recognized for its involvement in the presymptomatic stages of AD [67,68], with the degeneration of the inferior parietal lobule being especially detrimental for AD [69,70,71]. Therefore, plasma IGF-1 should be further assessed as a potential biomarker for these features, which aligns with the rising interest of using the atrophy of a composite of brain regions other than the hippocampus in isolation to aid the early diagnosis of AD [60,71].

By contrast to the findings for reduced brain volume measurements, we did not observe any significant changes in plasma IGF-1 levels in participants with significant CeVD burden in this study cohort. Although previous studies have highlighted IGF-1 involvement in white matter protection [72] or reduced WMH volumes [27,73], we also did not observe any IGF-1 association in WMH volumes in this cohort after adjustment for covariates (Table 3). Additionally, this study is, as far as we know, the first to investigate plasma IGF-1 associations with lacunes and microbleeds in a clinical cohort, and we again report no associations between IGF-1 and these small vessel disease subtypes, nor with larger cortical infarcts. As the cohort investigated by the present study is known to have high CeVD burden concomitant to amyloid pathology [74], our results point to the involvement of IGF-1 deficits in reduced brain volumes, while showing a possible lack of direct pathophysiological link to cerebrovascular-related pathology. This also suggests that plasma IGF-1 may be a useful biomarker in differentiating AD- from vascular cognitive impairment-driven processes. Furthermore, our results point to IGF-1 as a potential therapeutic target which may have protective mechanisms independent of its effect on glucose metabolism, since its association with brain atrophy remained significant after adjusting for diabetes mellitus (see Table 2). However, studies proposing intervention with IGF-1 may need to contend with a complex relationship between IGF-1 and brain neuroimaging or cognitive outcomes. For example, Cao et al. reported a U-shaped relationship between IGF-1 concentrations and risk of dementia and stroke in the UK Biobank Cohort [27], findings supported by a meta-analysis showing that mid-range IGF-1 concentrations in the body may be optimal for preventing cognitive decline [75]. This necessitates further studies elucidating the relationships amongst IGF-1, brain volumes and cognitive function.

The significant correlations observed between IGF-1 and both structural measures and cognitive outcomes suggest that IGF-1 may be related to both the structural and functional aspects of brain health. The correlation between IGF-1 and cognitive performance is consistent with the established relationship between brain structural changes and cognitive decline and may reflect the role of IGF-1 in neuronal survival, synaptic plasticity, and maintenance of brain structural integrity. Together, the observed associations support a potential link between higher IGF-1 levels, greater brain volumes, and better cognitive function.

The strengths of this study would be the careful clinical diagnoses of cognitive impairment and dementia based on comprehensive neuropsychological assessments, as well as the use of 3T-MRI to grade and classify participants with CeVD and brain atrophy. Furthermore, quantitative volumetric measurements for multiple individual brain regions were obtained using advanced neuroimaging platforms, enabling assessment of structural changes beyond the hippocampus. However, several limitations of this study should also be recognized. First, the cross-sectional study design precludes determination of temporal direction or causality of the observed associations between plasma IGF-1, structural brain measures and CeVD. While reduced regional volume and cortical thickness are commonly interpreted as imaging correlates of brain atrophy, longitudinal MRI measurements are required to directly quantify the rate and progression of atrophy. Therefore, we cannot determine whether lower IGF-1 precedes, accompanies, or results from the observed structural brain changes. Longitudinal studies incorporating repeated measurements of plasma IGF-1, cognitive outcomes, and MRI measures are needed to clarify the temporal relationship between IGF-1 and neurodegenerative and cerebrovascular changes. Next, plasma IGF-1 levels may be influenced by age and comorbid conditions, including hypertension, diabetes mellitus, metabolic syndrome, and cardiovascular disease, which were more prevalent in the AD group. Although these factors were statistically adjusted for in our analyses, residual confounding cannot be excluded. Thirdly, as the field moves towards a biomarker-based definition of AD, the association of IGF-1 levels with other pathological hallmarks of AD, such as amyloid and tau pathology, warrants further examination and validation from larger cohorts. Finally, although IGF-1 is one of the key proteins of the IGF signaling axis, potential confounding effects of other important components of the putative underlying pathways, such as IGF-2, IGF binding proteins (IGFBPs) and receptors, were not considered in this study. In particular, the associations between IGF-1 and IGFBPs should be further investigated given that different IGFBP subtypes differentially modulate IGF-1 bioavailability, stability, and receptor signaling [76].

## 5. Conclusions

This study demonstrates that decreased plasma IGF-1 is a potential biomarker for widespread structural brain changes, while showing no association with concomitant CeVD, in a cohort of participants with AD dementia. IGF-1 may be a therapeutic target for AD-associated neurodegeneration. Future studies are needed to validate the current findings and elucidate the pathophysiological mechanisms underlying the associations between IGF-1 deficits and brain atrophy.

## Figures and Tables

**Table 1:** Baseline demographics and neuroimaging variables of study participants.

	NCI	CIND	AD	*p*-Value
Maximum n	46	101	81	
*Demographics*				
Age, y	66 (6)	71 (9) ^a^	77 (7) ^a,b^	*
Female, n (%)	23 (50)	44 (43.6)	53 (65.4) ^b^	*
Education, y	10 (4)	7 (5) ^a^	4 (4) ^a,b^	*
APOE ε4 carrier, n (%)	11 (23.9)	29 (28.7)	29 (35.8)	n.s.
Hypertension, n (%)	28 (60.9)	73 (72.3)	67 (82.7) ^a^	*
Hyperlipidemia, n (%)	27 (58.7)	77 (76.2)	57 (70.4)	n.s.
Diabetes mellitus, n (%)	11 (23.9)	36 (35.6)	38 (46.9) ^a^	*
Cardiovascular disease, n (%)	3 (6.5)	19 (18.8)	15 (18.5)	n.s.
*Brain atrophy markers*				
Hippocampus volume, mL	6.6 (0.6)	6.1 (0.9) ^a^	5.0 (1.1) ^a,b^	*
Global cortical thickness, mm	2.4 (0.1)	2.3 (0.1) ^a^	2.2 (0.1) ^a,b^	*
Amygdala volume, mL	3.6 (0.3)	3.4 (0.5)	3.0 (0.6) ^a,b^	*
Frontal lobe volume, mL	149.2 (12.6)	138.1 (18.8) ^a^	123.8 (15.9) ^a,b^	*
Temporal lobe volume, mL	99.9 (8.7)	92.4 (13.3) ^a^	80.1 (10.8) ^a,b^	*
Parietal lobe volume, mL	72.7 (7.4)	68.0 (10.3) ^a^	60.9 (8.9) ^a,b^	*
Gray matter volume, mL	598.3 (45.3)	563.6 (61.3) ^a^	511.0 (49.2) ^a,b^	*
Total intracranial volume, mL	1434.4 (116.8)	1422.3 (131.9)	1370.6 (115.7) ^a,b^	*
*CeVD markers*				
Significant CeVD, n (%)	9 (19.6)	55 (55.0) ^a^	45 (57.0) ^a^	*
Cortical infarcts	0 (0)	0 (0)	0 (0)	n.s.
Lacunes	0 (0)	0 (1) ^a^	0 (1)	*
Cerebral microbleeds	0 (1)	0 (1)	1 (2)	*
ARWMC scores	4 (2)	6 (6) ^a^	7 (6) ^a,b^	*
WMH volume, mL	3.3 (5.1)	6.7 (17.1) ^a^	14.1 (19.0) ^a,b^	*
*Blood biomarker*				
Plasma IGF-1, ng/mL	65.6 (15.1)	61.2 (19.1)	49.2 (17.1) ^a,b^	*

Baseline demographics and neuroimaging variables of study participants. * denotes significant *p*-values (<0.05). Global cortical thickness data, hippocampal volume, amygdala volume, frontal lobe volume, temporal lobe volume, parietal lobe volume, gray matter volume, and intracranial volume data were available for 223 participants. WMH volume data were available for 206 participants. ARWMC score data were available for 224 participants. Cortical infarcts and lacunes data were available for 226 participants. CMB data were available for 217 participants. Abbreviations: AD, Alzheimer’s disease; ARWMC, Age-Related White Matter Changes; CeVD, cerebrovascular disease; CIND, cognitive impairment, no dementia; IGF-1, insulin-like growth factor 1; NCI, no cognitive impairment; WMH, white matter hyperintensity. ^a^ Significantly different from NCI; ^b^ Significantly different from CIND.

**Table 2:** Associations of plasma IGF-1 with regional brain volumes.

Plasma IGF-1 *	*Hippocampal* *Volume (n = 223)*	*Global Cortical Thickness (n = 223)*	*Amygdala Volume* *(n = 223)*	*Temporal Lobe Volume (n = 223)*	*Parietal Lobe* *Volume (n = 223)*	*Frontal Lobe* *Volume (n = 223)*	*Gray Matter Volume (n = 223)*
	β (95% CI)	β (95% CI)	β (95% CI)	β (95% CI)	β (95% CI)	β (95% CI)	β (95% CI)
Model 1	**1.1** **(0.2–2.0) ^# ^^**	**0.1** **(0–0.2) ^# ^^**	**0.5** **(0.1–1.0) ^# ^^**	**10.1** **(1.2–19.0) ^# ^^**	**9.3** **(1.9–16.7) ^# ^^**	**13.5** **(1.0–26.0) ^# ^^**	**43.8** **(11.5–76.0) ^# ^^**
Model 2	**1.0** **(0.1–1.8) ^# ^^**	**0.1** **(0–0.2) ^# ^^**	**0.5** **(0.1–0.9) ^# ^^**	8.6 (−0.4–17.7)	**8.3** **(0.8–15.8) ^# ^^**	**12.6** **(0.1–25.1) ^# ^^**	**39.5** **(7.5–71.5) ^# ^^**

Associations of plasma IGF-1 with regional brain volumes. Associations between plasma IGF-1 and brain volumes are expressed as β = mean difference with 95% CI. Bold font and **^#^** denote statistically significant associations and **^^^** denotes statistically significant associations following Benjamini–Hochberg FDR correction. Global cortical thickness, hippocampal, amygdala, temporal lobe, parietal lobe, frontal lobe and gray matter volumes data were not available for five participants. * Log-transformed. Model 1: Adjusted for age, sex, education and total intracranial volume. Model 2: Adjusted for age, sex, education, total intracranial volume, APOE4 status, hypertension, hyperlipidemia, diabetes mellitus and cardiovascular diseases. Abbreviations: CI, confidence interval; IGF-1, insulin-like growth factor 1.

**Table 3:** Associations of plasma IGF-1 with CeVD neuroimaging markers.

Plasma IGF-1 *	*Cortical Infarct Count* *(n = 226)*	*Lacune Count* *(n = 226)*	*CMB Count* *(n = 217)*	*ARWMC Score ≥ 8* *(n = 224)*		*WMH Volume* *(n = 206)*
	RR (95% CI)	RR (95% CI)	RR (95% CI)	OR (95% CI)		β (95% CI)
Model 1	0.6 (0.04–8.2)	1.0 (0.2–6.0)	0.5 (0.1–1.7)	1.7 (0.2–13.6)	Model 3	−10.2 (–24.7–4.4)
Model 2	0.3 (0.02–5.4)	0.7 (0.1–4.9)	0.6 (0.2–2.4)	1.7 (0.2–14.8)	Model 4	−9.7 (–24.6–5.2)

Associations of plasma IGF-1 with CeVD neuroimaging markers. Associations between plasma IGF-1 and cortical infarct counts, lacune count and CMB count are expressed as RR values with 95% CI. Associations between plasma IGF-1 and ARWMC scores are expressed as OR values with 95% CI. Associations between plasma IGF-1 and WMH volume are expressed as β = mean difference with 95% CI. Cortical infarct count and lacune count data was missing for two participants, CMB count data was missing for 11 participants, ARWMC score data was missing for four participants and WMH volume data was missing for 22 participants. * Log-transformed. Model 1: Adjusted for age, sex and education. Model 2: Adjusted for age, sex, education, hypertension, hyperlipidemia, diabetes mellitus and cardiovascular diseases. Model 3: Adjusted for age, sex, education and total intracranial volume. Model 4: Adjusted for age, sex, education, total intracranial volume, hypertension, hyperlipidemia, diabetes mellitus and cardiovascular diseases. Abbreviations: ARWMC, Age-Related White Matter Changes; CeVD, cerebrovascular disease; CI, confidence interval; IGF-1, insulin-like growth factor 1; OR, odds ratio; RR, rate ratio; WMH, white matter hyperintensities.

## Data Availability

Anonymized data derived from this study may be provided by the corresponding authors upon reasonable request.

## Supplementary Materials

The following supporting information can be downloaded at: https://www.mdpi.com/article/10.3390/biom16091248/s1, Table S1: Summary of Neuropsychological test battery and Component Tests; Data S1: Magnetic resonance imaging protocol and CeVD description; Table S2: Correlations of plasma IGF-1 with regional brain volumes; Table S3: Baseline demographics and neuroimaging characteristics of the participants excluding participants with large structural lesions on MRI scans (n = 7); Table S4: Associations of plasma IGF-1 with regional brain volumes excluding participants with large structural lesions on MRI scans (n = 7); Table S5: Associations of plasma IGF-1 with MRI markers of CeVD excluding participants with large structural lesions on MRI scans (n = 7); Table S6: Correlations of plasma IGF-1 with cognitive test scores; Figure S1: Plasma IGF-1 levels across diagnostic groups excluding participants with large structural lesions on MRI scans (n = 7).
