# Supplementary material for: Decreased Plasma IGF-1 Is Associated with Cortical Atrophy, but Not Concomitant Cerebrovascular Disease in Alzheimer’s Dementia
Source: Biomolecules. 2026 Aug 28;16(9):1248. doi: 10.3390/biom16091248 (PMC13604395; doi:10.3390/biom16091248)
Supplement: Supplementary file 1 [file biomolecules-16-01248-s001.zip › biomolecules-4427373-supplementary.pdf]

Yam *et al.*, Decreased plasma IGF-1 is associated with cortical atrophy, but not concomitant cerebral small vessel disease in Alzheimer's dementia

Supplementary Materials

Supplementary Table S1. Summary of Neuropsychological test battery and Component Tests

The following neuropsychological test battery was administered to assess cognitive function. The following seven (5 non-memory and 2 memory) domains were examined:

Cognitive Domain	Component Test(s)
Executive Function:	Frontal Assessment Battery [1]
Attention:	Digit Span, Visual Memory Span [2] and Auditory Detection [3]
Language:	Modified Boston Naming Test [4] and Verbal Fluency [5]
Visuomotor Speed:	Symbol Digit Modality Test [6, 7], Maze Task [7] and Digit Cancellation [8]
Visuoconstruction:	Weschler Memory Scale – Revised (WMS-R) Visual Reproduction Copy task [2], Clock Drawing [9] and Weschler Adult Intelligence Scale – Revised (WAIS-R) subtest of Block Design [10]
Visual Memory:	Picture Recall & Recognition Tasks, and WMS-R Visual Reproduction Recall & Recognition Task [2]
Verbal Memory:	Word List Recall & Recognition Tasks [11] and Story Recall Task

1. Dubois, B., et al., *The FAB: a Frontal Assessment Battery at bedside.* Neurology, 2000. **55**(11): p. 1621–1626.
2. Wechsler, D., *Wechsler Memory Scale-Revised*. 3rd ed. 1997, San Antonio, TX: Jovanovich.
3. Lewis, R.F. and P.M. Rennick, *Manual for the Repeatable Cognitive Percpetual-Motor Battery*. 1979, Clinton Township, MI: Axon.
4. Mack, W.J., et al., *Boston Naming Test: shortened versions for use in Alzheimer's disease.* Journal of Gerontology, 1992. **47**(3): p. P154–158.
5. Isaacs, B. and A.T. Kennie, *The Set test as an aid to the detection of dementia in old people.* The British Journal of Psychiatry: The Journal of Mental Science, 1973. **123**(575): p. 467–470.
6. Smith, A., *Symbol Digit Modalities Test*. 1973, Los Angeles, CA: ?
7. Porteus, S.D., *The Maze Test and Clinical Psychology*. 1959, Palo Alto, CA: Pacific Books.
8. Diller, L., Y. Ben-Yishay, and L.J. Gerstman, *Studies in Cognition and Rehabilitation in Hemiplegia*. 1974, New York: New York University Medical Center Institute of

Rehabilitation Medicine.
9. Sunderland, T., et al., *Clock drawing in Alzheimer's disease. A novel measure of dementia severity.* Journal of the American Geriatrics Society, 1989. **37**(8): p. 725–729.
10. Wechsler, D., *Wechsler Adult Intelligence Scale-Revised*. 1981, San Antonio, TX: Harcourt Brace Jovanovich.
11. Sahadevan, S., et al., *Cognitive testing of elderly Chinese people in Singapore: influence of education and age on normative scores.* Age and Ageing, 1997. **26**(6): p. 481–486.

Supplementary Data S1. Magnetic resonance imaging protocol and CeVD description

MRI protocol

The standardized protocol included a three-dimensional (3D) T1-weighted (1.0 X 1.0 X 1.0 mm3 voxels; repetition time (TR) 2300 ms; echo time (TE) 1.9 ms; inversion time (TI) 900 ms; flip angle 9°; matrix 256 X 256), a 2D multislice T2-weighted (1.0 X 1.0 X 3.0 mm3 voxels; TR 3000 ms; TE 10.1 ms; matrix 247 X 256), a 2D multislice FLAIR (1.0 X 1.0 X 3.0 mm3; TR 9000 ms; TE 82 ms; TI 2500 ms; matrix 232 X 256), and a 2D multislice T2*-weighted image (1.0 X 1.0 X 1.5 mm3 voxels; TR 27 ms; TE 20 ms; flip angle 15°; matrix 192 X 256), for the assessment of markers of small vessel disease (white matter hyperintensities and lacunes).

MRI Visual gradings: Lacunes, cerebral microbleeds (CMBs), cortical infarcts

Lacunes were defined as lesions, 3-15mm in diameter, with low signal on T1 weighted image and FLAIR; a high signal on T2 weighted image, and a hyperintense rim with a center following CSF intensity on FLAIR. CMBs were graded using the Brain Observer Microbleed Scale and were defined as focal, rounded areas of hypointensity, 2-10mm in diameter, with blooming on SWI. The total number of CMBs in the lobar and deep regions was recorded and used. Cortical infarcts were defined as focal lesions involving cortical grey matter, a signal following CSF intensity, a hyperintense rim on FLAIR images, and tissue loss of variable magnitude, with prominent adjacent sulci and ipsilateral ventricular enlargement.

Supplementary Table S2. Correlations of plasma IGF-1 with regional brain volumes

Brain Region	*n*	*IGF-1 Spearman's rho*
Hippocampus	223	**0.351***
Global Cortical Thickness	186	**0.327***
Amygdala	223	**0.328***
Temporal Lobe	223	**0.365***
Parietal Lobe	223	**0.370***
Frontal Lobe	223	**0.374***
Gray Matter	223	**0.389***

Spearman's correlations between plasma IGF-1 concentrations and MRI measures of regional brain volumes. *Represents significant *p*-values (<0.05).

Supplementary Table S3. Baseline demographics and neuroimaging characteristics of the participants excluding participants with large structural lesions on MRI scans (n=7)

	NCI	*CIND*	*AD*	*p-value*
Maximum n	45	98	78	
Demographics				
Age, y	66 (6)	71 (9) [a]	77 (7) [a,b]	*
Female, n (%)	23 (51.1)	44 (44.9)	52 (66.7) [b]	*
Education, y	10 (4)	7 (5) [a]	4 (4) [a,b]	*
APOE ε4 carrier, n (%)	11 (24.4)	28 (28.6)	29 (37.2)	n.s.
Hypertension , n (%)	27 (60.0)	70 (71.4)	64 (82.1) [a]	*
Hyperlipidaemia, n (%)	26 (57.8)	74 (75.5)	54 (69.2)	n.s.
Diabetes Mellitus, n (%)	11 (24.4)	35 (35.7)	38 (48.7) [a]	*
Cardiovascular diseases, n (%)	2 (4.4)	16 (16.3)	13 (16.7)	n.s.
Brain atrophy				
Hippocampus volume, mL	6.7 (0.6)	6.1 (1.0) [a]	5.0 (1.0) [a,b]	*
Global cortical thickness, mm	2.4 (0.1)	2.3 (0.1) [a]	2.2 (0.1) [a,b]	*
Amygdala volume, mL	3.6 (0.3)	3.4 (0.5)	3.0 (0.6) [a,b]	*
Frontal lobe volume, mL	149.5 (12.6)	138.2 (18.9) [a]	123.5 (16.0) [a,b]	*
Temporal lobe volume, mL	99.6 (8.6)	92.5 (13.5) [a]	79.8 (10.9) [a,b]	*
Parietal lobe volume, mL	72.6 (7.4)	68.2 (10.3) [a]	61.0 (9.0) [a,b]	*
Gray matter volume, mL	597.4 (45.4)	563.6 (62.1) [a]	510.5 (49.2) [a,b]	*
Total intracranial volume, mL	1431.4 (116.4)	1420.4 (133.5)	1370.1 (115.8) [a,b]	*
CeVD markers				
Cortical infarcts	0 (0)	0 (0)	0 (0)	n.s.
Lacunes,	0 (0)	0 (1) [a]	0 (1)	*
Cerebral microbleeds	0 (1)	0 (1)	0 (2) [a]	*
ARWMC scores	4 (3)	6 (6) [a]	8 (6) [a,b]	*
WMH volume, mL	3.1 (5.1)	6.4 (17.1) [a]	13.5 (20.0) [a,b]	*
Biomarker				
Plasma IGF-1, ng/ml, mean (SD)	65.9 (15.1)	61.0 (19.3)	48.9 (17.1) [a,b]	*

*Denotes significant p-values. Global cortical thickness data, hippocampal volume, amygdala volume, temporal lobe volume, parietal lobe volume, gray matter volume, intracranial volume data was available for 216 participants. WMH volume data was available for 199 participants. ARWMC scores data was available for 217 participants. Cortical infarcts and lacunes data were available for 219 participants. CMBs data was available for 210 participants. Abbreviations: AD, Alzheimer's disease; ARWMC, Age-related White Matter Changes; CeVD, cerebrovascular disease; CIND, cognitive impairment, no dementia; IQR, inter-quartile range; IGF-1, insulin-like growth factor 1; NCI, no cognitive impairment; SD, standard deviation; WMH, white matter hyperintensity. [a] Significantly different from NCI (one-way ANOVA with post hoc Bonferroni tests $p < 0.05$). [b] Significantly different from CIND (one-way ANOVA with post hoc Bonferroni tests $p < 0.05$).

Supplementary Table S4. Associations of plasma IGF-1 with regional brain volumes excluding participants with large structural lesions on MRI scans (n = 7)

*Plasma IGF-1**	*Hippocampal volume (n = 216)*	*Global cortical thickness (n = 216)*	*Amygdala volume (n = 216)*	*Temporal lobe volume (n = 216)*	*Parietal lobe volume (n = 216)*	*Frontal lobe volume (n = 216)*	*Gray matter volume (n = 216)*
	β (95% CI)	β (95% CI)	β (95% CI)	β (95% CI)	β (95% CI)	β (95% CI)	β (95% CI)
Model 1	**1.1 (0.3-2.1)# ^**	**0.2 (0.1-0.4)# ^**	**0.5 (0.1-1.0)# ^**	**10.6 (1.5-19.7)# ^**	**8.8 (1.4-16.2)# ^**	**12.9 (0.4-25.5)# ^**	**45.0 (12.2-77.8)# ^**
Model 2	**1.0 (0.1-1.9)#**	**0.1 (0-0.2)#**	**0.5 (0-0.9)#**	8.9 (-0.3-18.1)	**7.6 (0-15.1)#**	11.9 (-0.7-25.0)	**39.7 (6.9-72.4)#**

Associations between plasma IGF-1 and brain volumes are expressed as β = mean difference with 95% CI. Bold font and # denotes statistically significant associations and ^ denotes statistically significant associations following Benjamini-Hochberg FDR correction. Global cortical thickness, Hippocampal, temporal lobe, parietal lobe, frontal lobe and gray matter volumes data was missing for 5 participants. *Log-transformed. *Model 1*: Adjusted for age, sex, education and total intracranial volume. *Model 2*: Adjusted for age, sex, education, total intracranial volume, APOE4 status, hypertension, hyperlipidemia, diabetes mellitus and cardiovascular diseases. Abbreviations: CI, confidence interval; IGF-1, insulin-like growth factor 1.

Supplementary Table S5. Associations of plasma IGF-1 with MRI markers of CeVD excluding participants with large structural lesions on MRI scans (n = 7)

*Plasma IGF-1**	*Cortical infarcts count (n = 219)*	*Lacunes count (n = 219)*	*CMBs count (n = 210)*	*ARWMC score ≥ 8 (n = 217)*		*WMH volume (n = 199)*
	RR (95% CI)	RR (95% CI)	RR (95% CI)	OR (95% CI)		β (95% CI)
Model 1	0.1 (0-2.9)	1.1 (0.2-6.8)	0.8 (0.2-2.8)	1.6 (0.2-12.9)	*Model 3*	-10.9 (-25.9-4.1)
Model 2	0.1 (0-2.5)	0.8 (0.1-5.6)	0.9 (0.2-3.6)	1.7 (0.2-14.7)	*Model 4*	-10.1 (-25.4-5.2)

Associations between plasma IGF-1 and cortical infarct counts, lacunes count and CMBs count are expressed as RR values with 95% CI. Associations between plasma IGF-1 and ARWMC scores are expressed as OR values with 95% CI. Associations between plasma IGF-1 and WMH volume are expressed as β = mean difference with 95% CI. Cortical infarcts count and lacunes count data was missing for 2 participants, CMB counts data was missing for 11 participants, ARWMC scores data was missing for 4 participants and WMH volume data was missing for 22 participants. *Log-transformed. *Model 1*: Adjusted for age, sex and education. *Model 2*: Adjusted for age, sex, education, hypertension, hyperlipidemia, diabetes mellitus and cardiovascular diseases. *Model 3*: Adjusted for age, sex, education and total intracranial volume. *Model 4*: Adjusted for age, sex, education, total intracranial volume, hypertension, hyperlipidemia, diabetes mellitus and cardiovascular diseases. ns, non-significant p values (>0.05). Abbreviations: ARWMC, Age-related White Matter Changes; CeVD, cerebrovascular disease; CI, confidence interval; CMBs, cerebral microbleeds; IGF-1, insulin-like growth factor 1; OR, odds ratio; RR, rate ratio; WMH, white matter hyperintensities.

Supplementary Table S6. Correlations of plasma IGF-1 with cognitive test scores

Cognitive test	*n*	*IGF-1 Spearman's rho*
MMSE	228	**0.245***
CDR-SoB	227	**-0.277***

Spearman's correlations between plasma IGF-1 concentrations and cognitive test scores. *Represents significant *p*-values (<0.05). Abbreviations: IGF-1, insulin-like growth factor 1; MMSE, Mini-Mental State Examination; CDR-SoB, Clinical Dementia Rating-Sum of Boxes.

Supplementary Figure S1. Plasma IGF-1 levels across diagnostic groups excluding participants with large structural lesions on MRI scans

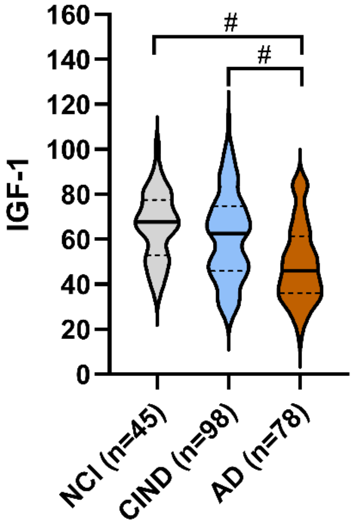

Plasma IGF-1 concentrations (in ng/mL) across diagnostic groups based on clinical diagnosis after removal of n=7 participants with large cortical infarcts, depicted in violin graphs with the middle solid lines denoting median values, while top and bottom dotted lines represent respective quartiles. Abbreviations: AD, Alzheimer's Disease; CIND, cognitively impaired no dementia; NCI, no cognitive impairment. $^{\#}p < 0.05$ (Kruskal-Wallis ANOVA with *post-hoc* Dunn-Bonferroni's tests).
